# Cues of Trained Immunity in Multiple Sclerosis Macrophages

**DOI:** 10.3390/cells14141054

**Published:** 2025-07-10

**Authors:** Elisa Popa, Hélène Cheval, Violetta Zujovic

**Affiliations:** Institut du Cerveau—Paris Brain Institute—ICM, Inserm, CNRS, APHP, Hôpital Pitié Salpêtrière University Hospital, Sorbonne Université, DMU Neuroscience 6, Paris, France

**Keywords:** multiple sclerosis, macrophages, trained immunity, metabolism, epigenetic, inflammation

## Abstract

Multiple sclerosis (MS) is a complex autoimmune disease with both genetic and environmental influences, yet its underlying mechanisms remain only partially understood. In this review, we compile evidence suggesting that trained immunity—a form of innate immune memory—may play a crucial role in the autoimmune component of MS. By examining key findings from immunology, neuroinflammation, and MS pathophysiology, we explore how innate immune cells, particularly monocytes and macrophages, could contribute to disease onset and progression through persistent pro-inflammatory responses. Understanding the impact of trained immunity in MS could open new avenues for therapeutic strategies targeting the innate immune system.

## 1. Introduction

The immune system encounters numerous inputs, ranging from infections (caused by viruses and bacteria) to environmental factors and vaccinations. Traditionally, immune memory has been attributed to adaptive immunity, where T and B cells retain antigen-specific recall for rapid, robust responses upon re-exposure. However, emerging research reveals that innate immune cells, such as monocytes and macrophages, also exhibit memory-like properties through a process termed “trained immunity”. This phenomenon, first identified with the Bacille Calmette-Guérin (BCG) vaccine [1], involves the long-term functional reprogramming of innate cells, enabling heightened responses to both homologous and heterologous pathogens (cross-protection). BCG vaccination has shown protective effects beyond its primary purpose of tuberculosis prevention, offering non-specific protection against infections such as *Staphylococcus aureus*, yellow fever, and even COVID-19 [2,3,4]. This non-specific protection stems from epigenetic and metabolic reprogramming in monocytes and macrophages, which results in an enhanced pro-inflammatory response upon re-exposure to pathogens.

While advantageous against infections, this heightened responsiveness can become detrimental in chronic inflammatory contexts, such as atherosclerosis [5,6], type 1 diabetes, lupus, and potentially multiple sclerosis (MS) [4], where it may amplify tissue damage.

## 2. Mechanisms of Trained Immunity

The heightened response of innate immune cells is driven by two key biological processes: metabolic changes and epigenetic reprogramming, which lead to the increased production of inflammatory mediators.

### 2.1. Metabolic Shift

Several studies have shown that trained immunity entails a metabolic reprogramming in monocytes and macrophages—shifting from oxidative phosphorylation (OXPHOS) toward aerobic glycolysis in a manner reminiscent of the cancer cell “Warburg effect” [7]. This switch accelerates ATP generation, boosts reactive oxygen species production, and supplies biosynthetic intermediates, all of which fuel a heightened pro-inflammatory phenotype and enable rapid pathogen clearance.

When β-glucan—a component of the *Candida albicans* cell wall—is used to trigger trained immunity, human monocytes increase glucose uptake and convert it to lactate via the AKT-mTOR-HIF1α pathway to support a surge in glycolysis [8]. Parallel work in atherosclerotic mice—in which inflammation overlaps mechanistically with MS—demonstrates that HIF1α-positive macrophages show enhanced glycolysis and cytokine outputs, reinforcing their pro-inflammatory identity in vivo and in vitro [9]. Remarkably, even hematopoietic stem cells (HSCs)—the precursors of circulating monocytes and macrophages—exhibit elevated glucose metabolism, suggesting that metabolism priming begins at the progenitor stage and may be inherited by their monocytes’/macrophage’s progeny [10]. Thus, the interplay between metabolic alterations at the progenitor level and trained immunity in monocytes and macrophages may create a continuum that amplifies inflammation.

However, the relationship between glycolysis and OXPHOS in trained immunity is far more nuanced than a simple either/or [11]. Rather than fully abandoning mitochondrial respiration, trained cells often co-activate both pathways: some studies report an early rise in OXPHOS [12], evidenced by higher NAD^+^/NADH ratios and ATP output, while others note increased oxygen consumption alongside a reduced reliance on OXPHOS for ATP generation [8]. This dual engagement provides metabolic flexibility, allowing mitochondria to channel tricarboxylic acid (TCA) cycle intermediates (e.g., succinate, fumarate) into biosynthesis and epigenetic regulation, even as glycolysis fuels immediate energy needs [13,14]. Recently, Cai et al. revealed that in trained monocytes of human and mice, lactate serves as a preferred substrate for the TCA cycle even in the presence of glucose [15]. This utilization of lactate enhances acetyl-CoA production, supplying the TCA cycle and supporting the sustained production of pro-inflammatory cytokines.

### 2.2. Epigenetic Reprogramming

Epigenetics refers to heritable changes in gene expression that do not involve alterations to the underlying DNA sequence and are influenced by environmental factors. These changes, such as DNA methylation and histone modifications, regulate DNA accessibility and control the activation or repression of genes. These modifications are pivotal in trained immunity, as they modify chromatin structure to improve accessibility for transcription factors.

Quintin et al. showed that β-glucan stimulates trained immunity in healthy donors’ monocytes and mouse macrophages, which is associated with an increase in activating histone marks such as H3K4me3 at the promoters of inflammatory genes [16]. Further studies elucidated that macrophage priming is supported by modifications of histone marks, including H3K4me1 (at distal regulatory elements such as enhancers), H3K4me3 (at promoters), and H3K27ac (at active promoters and enhancers), which enhance chromatin accessibility; this is demonstrated by an increased susceptibility to DNase I. These findings show that trained immunity’s induction is not restricted to promoters but encompasses a larger epigenetic landscape, in which distal regulatory elements and active enhancers take part [17].

In addition to DNA’s regulatory elements, non-coding transcripts, such as long non-coding RNAs (lncRNAs) became a focus of interest because of their multiple regulatory functions, from the epigenetic to the post-translational levels, and for their involvement in crucial developmental and cellular processes, such as cell differentiation and maturation. As a corollary, a growing body of literature associates lncRNAs with a large variety of human diseases, such as cancer or neurological diseases [18,19]. In this context, Fanucchi et al. identified a lncRNA, termed *UMLILO* for “Upstream master lncRNA of the inflammatory chemokine locus”, that recruits the WDR5-MLL1 complex to this genomic region in trained human monocytes. This complex brings in MLL1, a histone methyltransferase, which adds an H3K4me3 mark at the chemokines’ promoter, loosening the chromatin and thus increasing gene accessibility and expression. This mark is long-lasting; following BCG exposure, *UMLILO* activation establishes a trained immune state, allowing inflammatory genes to respond faster and more strongly to secondary infections [20].

These durable epigenetic changes underpin the long-term reactivity of trained cells [21].

### 2.3. Metabolo-Epigenetics

Trained immunity is characterized by the durable, functional reprogramming of innate immune cells, underpinned by intricate interactions between metabolic pathways and epigenetic modifications.

A pivotal aspect of this reprogramming involves, as stated above, the accumulation of lactate. It serves not only as a metabolic substrate but also as an epigenetic modulator. Specifically, lactate promotes histone lactylation, increasing chromatin accessibility and facilitating the transcription of pro-inflammatory cytokines such as *Il-6* and *Tnf-α* [15]. This dual role of lactate underscores its centrality in bridging metabolic activity and gene expression during trained immunity establishment. The TCA cycle metabolites also contribute to epigenetic remodeling. Acetyl-CoA serves as a substrate for histone acetyltransferases, leading to histone acetylation. The H3K27ac mark at both promoters and enhancers has been associated with the induction of trained macrophages [17]. Furthermore, the accumulation of fumarate has been shown to inhibit histone demethylases such as KDM5. This inhibition results in increased levels of H3K4me3 at the promoters of *IL-6* and *TNF-α*, sustaining their elevated expressions during subsequent immune challenges [13].

Conversely, epigenetic modifications also influence metabolic pathways. In monocytes trained with β-glucan, there is an upregulation of glycolytic enzymes, including hexokinase 2 (HK2) and phosphofructokinase platelet (PFKP). This upregulation is associated with increased deposition of activating H3K4me3 marks at the promoters of these genes, enhancing their expressions and glycolytic activities [12].

This dynamic crosstalk between metabolic pathways and epigenetic regulation equips innate immune cells with an adaptive edge, enhancing their protective role over time [21].

### 2.4. Cytokine Expression

All these reprogramming events enhance monocytes’/macrophages’ inflammatory response upon subsequent encounters. This results in an increased secretion of pro-inflammatory cytokines, such as IL-1β, TNF-α, and IL-6, as well as chemokines, such as monocyte MCP-1, CXCL1, CXCL3, and others, upon restimulation [1,20,22]. These findings suggest that trained immunity equips innate immune cells with a form of memory, enabling a more robust response to secondary infections.

While protective against pathogens, these mechanisms can paradoxically be at the root of chronic inflammation in autoimmunity. This raises the question about their role in MS and how such persistent responses might be therapeutically modulated.

## 3. Trained Immunity in MS: A New Perspective

MS is a chronic condition in which the immune system attacks the central nervous system (CNS) and destroys one of its essential components: the myelin. This destruction is the consequence of the CNS invasion by both lymphocytes and macrophages, which contributes to myelin damage and can ultimately lead to neurodegeneration if not repaired. Interestingly, the CNS possesses a self-repair mechanism known as remyelination, a process also regulated by lymphocytes and macrophages. Macrophages play a dual role in this context: they can adopt a pro-inflammatory phenotype, which exacerbates myelin damage, or a pro-regenerative phenotype, which promotes remyelination. The transition between these phenotypes is finely regulated, underscoring the complex and dynamic nature of macrophage function in MS [23]. Here, different MS monocyte/macrophage aspects, reminiscent of trained immunity, are emphasized.

### 3.1. Monocyte Populations More Prone to Inflammation

**Figure 1:**
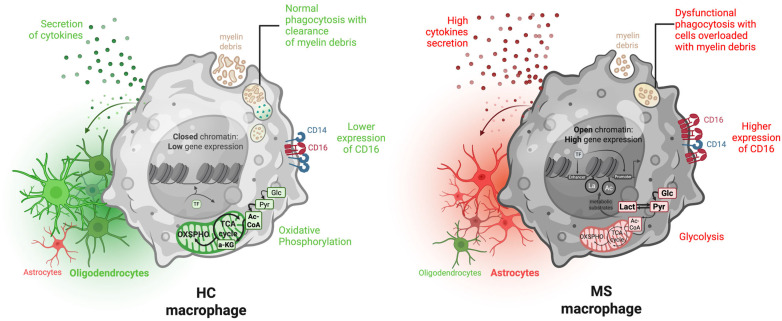
Functional and metabolic dysregulation of macrophages in MS patients and potential epigenetic rearrangements. On the left, macrophages from HCs exhibit balanced OXPHOS, effective phagocytosis with efficient clearance of myelin debris, and low CD16 expression. On the right, MS macrophages show increased CD16 expression, accumulation of myelin debris due to impaired phagocytosis, and a greater dependance on glycolysis and elevated cytokine secretion. This leads to a greater differentiation toward astrocytes rather than oligodendrocytes when glial progenitors are exposed to MS macrophages-conditioned media compared with HCs. This heightened inflammatory phenotype could be due to epigenetic rewiring (hypothesis illustrated in grey—Elisa). This scheme was created on Biorender https://biorender.com/.

Studies have shown that MS patients have a higher proportion of non-classical CD14^+^CD16^++^ monocytes in their blood compared with healthy controls (HCs) [24,25]. A similar increase is observed in the cerebrospinal fluid (CSF) of MS patients [26]. Chuluundorj et al. demonstrated that these non-classical CD16^+^ monocytes drive the inflammatory response in MS [27]. In a follow-up study, they showed that monocytes from untreated MS patients exhibit an overexpression of activation markers—such as CD40, CD86, HLA-DR, CD64, and CCR2—suggesting a heightened state of activation, and these cells are predominantly CD16^+^ [28]. Carstensen et al. further revealed that non-classical monocytes in MS patients exhibit higher expressions of the human endogenous retrovirus (HERV) H3 envelope epitope, indicating inflammatory activation [29]. Finally, our recent study showed that isolated CD14^+^CD16^−^ monocytes from MS patients are more likely to differentiate into CD16^+^ macrophages compared with HCs (Figure 1) [30]. This shift may contribute to chronic inflammation and heightened T cell activation, possibly driven by CD16-dependent antigen presentation in the context of MS.

### 3.2. Altered Metabolism

Recent research has illuminated metabolic differences in monocytes and macrophages subpopulations in MS. Using untargeted metabolomics analysis, Zahoor et al. revealed that peripheral blood mononuclear cells (PBMCs) from relapsing–remitting MS (RRMS) patients displayed heightened glycolysis compared with those from HCs [31]. They further studied the role of glycolysis and its modulation of disease progression in an animal model of MS: experimental autoimmune encephalomyelitis (EAE). They confirmed that PBMCs from EAE mice exhibited significantly higher glycolytic activity compared with the control group, and when they treated EAE mice with 2-deoxy-D-glucose (2DG), a known glycolytic inhibitor, they observed a delayed disease onset and reduced severity. Importantly, macrophages from 2DG-treated EAE mice polarized toward an anti-inflammatory phenotype characterized by the increased expression of *Arginase-1* and *Chil3/4* (Ym1/2) and the decreased expression of *Nos2* (commonly referred to as iNOS) and *Il-1β*. This shift was further validated by adoptive transfer experiments, in which transferring 2DG-treated monocytes into EAE mice ameliorated disease severity, underscoring the therapeutical potential of modulating monocytes’ metabolism. Additionally, macrophages from untreated MS patients showed decreased activity in energy-related metabolic processes, such as TCA cycle, fatty acid oxidation, and the electron transport chain, along with lower NAD^+^ levels, when compared with HCs and treated patients (Figure 1) [30]. This impaired mitochondrial function and altered glycolytic capacity could be the underlying cause of the abnormal over-inflammatory responses to activation stimuli observed in the macrophages of MS patients.

Analysis of single-cell RNA sequencing data uncovered notable variations in the activity of aromatic amino acid (AAA) metabolic pathways within blood- and cerebrospinal fluid-derived monocytes [32]. Specifically, the tryptophan and tyrosine catabolism pathways were downregulated, accompanied by altered levels of kynurenine and indolelactic acid (tryptophan metabolism) as well as of 3-(4-hydroxyphenyl) lactate and 4-hydroxyphenylpyruvate (tyrosine metabolism). This reduction coincided with an upregulation of oxidative products, such as p-cresol sulfate/glucuronide, indoleacetate, and phenylacetylglutamine. This imbalance change correlated strongly with disability levels, as assessed by the Expanded Disability Status Scale (EDSS) and Age-Related Multiple Sclerosis Severity (ARMSS). They further demonstrated that treatment with AAA-derived reductive metabolites could suppress monocytes’ pro-inflammatory features. While 4-hydroxyphenylpyruvate can generate TCA metabolites, it remains an open question whether the other AAA disruptions impair OXPHOS in MS macrophages, potentially affecting precursors or substrates such as acetyl-CoA, NADH, and FADH2. These pathways are essential for supporting a pro-regenerative macrophage phenotype.

### 3.3. Epigenetics Insight into MS

Ma et al. conducted a comprehensive study integrating epigenetic and genetic data to identify cell types and genes critical to MS pathogenesis [33]. Their analysis revealed that monocytes and microglia are central to MS development, with MS genetic variants enriched in their regulatory regions that contribute both to disease risk and severity, as evidenced by their impact on clinical outcomes like white matter volume. Similarly, Guo et al. demonstrated a strong association between regions of open chromatin in monocytes and genetic signals linked to MS, further emphasizing the importance of epigenetic modifications in the disease process [34]. Notably, Ma et al. identified genes, including *NFKB1*, *STAT3*, and *IRF8*, which regulate immune responses and are influenced by MS-associated variants [33]. These findings suggest that genetic variants may impact gene expression in monocytes and microglia, contributing to MS pathogenesis through altered immune function. Moreover, the altered chromatin accessibility in these cells may heighten their sensitivity to environmental triggers, such as infections or dietary factors, leading to epigenetic reprogramming and enhanced pro-inflammatory responses.

As mentioned before, lncRNAs are recognized as potent genomic regulators capable of modulating the epigenetic landscapes of cells. These molecules are of particular interest due to their high specificity to both cell types and species, making them promising therapeutic candidates in human pathologies characterized by immune cell dysfunctions such as MS [35,36]. Despite the relatively limited number of lncRNAs functionally characterized to date, some have already been implicated in the pathophysiology of MS. Han et al. investigated the role of lncRNAs in MS pathogenesis, particularly their interactions with MS-associated single-nucleotide polymorphisms (SNPs). Their findings indicate that SNP-driven regulation of lncRNAs primarily impacts antigen processing and presentation as well as the MAPK signaling pathway [37]. In parallel, Safa et al. explored the involvement of lncRNAs associated with the NF-κB signaling in MS and identified *MKI67IP* and *HNF1A-AS1* as key inhibitors of this pathway. These lncRNAs, known to inhibit the NF-κB pathway, were found to be downregulated in the blood cells of MS patients compared with HCs [38]. Given their role in modulating inflammatory responses, these lncRNAs represent potential targets for therapeutic intervention aimed at altering the inflammatory phenotype of MS-associated monocytes and macrophages.

Considering the significance of the non-coding genome and the higher likelihood of polymorphisms occurring in these regions, other non-coding RNA species, such as microRNAs (miRNAs), have also been investigated. Amoruso et al. conducted a study examining the expression profiles of miRNAs in MS monocytes and showed a significant upregulation of *miR-155* and a downregulation of *miR-223* in these cells that was associated with an imbalance in monocyte/macrophage polarization, favoring a pro-inflammatory state [39].

Overall, these findings highlight lncRNAs and miRNAs as promising therapeutic targets. Modulating their expression may provide a means to prevent or even reverse the persistent hyper-inflammatory state characteristic of MS-associated monocytes and macrophages.

### 3.4. Elevated Cytokine Secretion

Monocytes derived from the blood of MS patients exhibit increased secretion of inflammatory cytokines, including IL-6 and IL-12, relative to HCs [40]. Specifically, the CD16+ monocyte subpopulation in MS patients has been pinpointed as releasing higher levels of pro-inflammatory cytokines and aiding T cell migration across an in vitro endothelial barrier, a process significant in the pathology of MS [24]. Likewise, PBMCs from patients with primary progressive MS (PPMS) overexpress pro-inflammatory cytokines, including *IL-1β*, *IL-6*, *TNF*, and *NLRP3*, with monocytes being the primary driver of this profile [41]. Lastly, Fransson et al. found that MS macrophages overexpress pro-inflammatory cytokines such as *CCL4*, even under homeostatic and pro-regenerative conditions, while showing a reduced level of anti-inflammatory cytokines like *CCL17* as compared with HCs (Figure 1) [30].

Collectively, these studies underscore how metabolic, epigenetic, and genetic cues converge to drive immune dysregulation in MS (Table 1). Intriguingly, elements of trained immunity imply that the innate immune system in MS may retain a memory of prior exposures, skewing macrophage polarization toward either a pro-inflammatory or pro-regenerative phenotype. This phenomenon may account for patient-to-patient differences in remyelination [42], as an excessive pro-inflammatory response may impede effective repair, whereas a balanced or pro-regenerative profile could support myelin restoration. 

## 4. Risks Factors of Trained Immunity in MS

### 4.1. Epstein-Barr Virus (EBV)

Research indicates that EBV is strongly linked to MS, with Bjornevik et al.’s study showing that antibodies against EBV nuclear-antigen (EBNA-1) are associated with an increased risk for MS [43]. Their review suggests that EBV can infect HSCs. This infection could lead to trained immunity, where these cells are epigenetically reprogrammed to have enhanced pro-inflammatory responses, contributing to MS pathology. In contrast, the seroconversion to cytomegalovirus (CMV) in EBV-seropositive people has been associated with a lower risk of MS where antibodies against CMV were linked to a decreased MS risk. Notably, a presymptomatic case-control study by Grut et al. further supports this inverse association, demonstrating that CMV seropositivity was significantly less frequent among individuals who later developed MS as compared with matched HCs [44]. This association remained significant even after adjusting for EBV and HHV-6A serostatus, suggesting an independent protective effect of CMV. These findings point toward a potential immunomodulatory role of CMV in shaping innate immune memory.

The contrasting effects of EBV and CMV on MS risk underscore the importance of viral interactions in disease pathogenesis. EBV’s role in inducing the trained immunity that contributes to MS suggests potential therapeutic targets, such as modulating epigenetic reprogramming in monocytes or targeting EBV-specific immune responses. Conversely, CMV’s protective effect might inspire strategies to mimic its immunoregulatory properties such as inducing tolerance in innate immune cells to prevent autoimmunity.

### 4.2. Western Diet

MS is more prevalent in regions where the Western diet prevails—a diet marked by the high consumption of saturated fats, refined carbohydrates, red meat, sugar-sweetened beverages, and fried food, along with low fiber intake and a sedentary lifestyle. Riccio et Rossano noted a higher MS incidence in Westernized countries, potentially due to dietary influences on inflammation and immune function [45]. Similarly, another review highlights the fact that the growing adoption of a Western diet over recent decades correlates with increased MS incidence, suggesting the important role that environmental factors, such as diet, play [46].

Several studies emphasize the fact that childhood obesity—a condition often associated with this dietary regimen—may have a lasting impact on MS susceptibility. A growing body of evidence links pediatric obesity to higher MS risk. For instance, a nationwide study by Hagman et al. demonstrated that obese children and adolescents are at a significantly higher risk for MS, particularly when obesity occurs early or is severe [47]. Longitudinal studies have similarly shown that pediatric MS patients are more likely to follow elevated body mass index trajectories compared with HCs [48,49]. Two studies by Langer-Gould et al. and Munger et al. further revealed that a higher body mass index during childhood independently correlates with an increased risk of pediatric MS and clinically isolated syndrome (CIS), suggesting a possible dose–response effect [50,51]. These findings point to excess adiposity during key periods of immune system development as key contributors to neuroinflammation and autoimmunity, positioning obesity, shaped in part by diet, as a modifiable MS risk factor.

Mechanistically, studies have shown that high glucose levels can induce trained immunity in macrophages resulting in persistent pro-inflammatory phenotypes [52]. Hyperglycemia causes epigenetic reprogramming both in mouse and human immune cells, enhancing and fostering chronic inflammatory diseases such as atherosclerosis. A particularly relevant study used Ldr^−/−^ mice, a genetically modified model lacking the low-density lipoprotein receptor, that are highly susceptible to hyperlipidemia and systemic inflammation when fed a high-fat, Western-style diet. This study demonstrated that consumption of a Western diet induced systemic inflammation and long-lasting reprogramming of myeloid cell responses even after switching to a healthier diet [53]. These effects were mediated by the NLRP3 inflammasome, leading to sustained production of Il-1β and Il-18. Critically, the Western diet drove transcriptional and epigenetic reprogramming of myeloid progenitor cells, promoting their expansion and priming them for heightened inflammatory responses—a hallmark of trained immunity.

## 5. Therapeutic Implications: Harnessing Trained Immunity for Repair

MS prognosis is closely tied to the extent of lesions and remyelination, for which macrophages and microglia play a dual role: pro-inflammatory phenotypes promote oligodendrocyte precursor cells (OPCs) proliferation, while pro-regenerative phenotypes drive OPC differentiation [54]. The transition from pro-inflammatory to pro-regenerative macrophage phenotypes is essential for effective remyelination [55].

Recently, we demonstrated that MS macrophages exhibit intrinsic defects, showing a tendency to become more inflammatory and less regenerative even in the absence of inflammatory signals and prior to encountering the lesion context [30]. This suggests a baseline pro-inflammatory bias, potentially driven by trained immunity. In vitro, these macrophages induce greater differentiation of the OPC lineage into astrocytes compared with HCs, indicating their inflammatory function (Figure 1). Additionally, MS macrophages display phagocytosis dysfunction since they are less capable of ingesting human myelin as compared with HCs’ macrophages, and the ones that do phagocytose myelin remain highly loaded, suggesting an impaired ability to metabolize the ingested myelin (Figure 1). Such functional deregulation has been described in atherosclerosis [56]. The downregulation of phagocytosis in MS macrophages may lead to inadequate clearance of myelin debris, which is necessary for the recruitment and differentiation of new OPCs [57]. Collectively, these findings (including the lack of pro-inflammatory to pro-regenerative phenotype switching, an inflammatory secretory profile, and dysregulated phagocytosis) may explain the impaired remyelination capacity in MS patients.

In this context, a recent study by Tiwari et al. showed that in a murine model of demyelination, microglia respond to peripheral BCG stimulation by undergoing epigenetic reprogramming seen with histones’ modifications [58]. Thus, they showed that BCG stimulation as well as the direct inhibition of the Hdac1 and Hdac2 histone deacetylases’ activity, were sufficient to improve the recovery of demyelinated lesions in aged mice. Functionally, they enhanced myelin phagocytosis by trained microglia and promoted better differentiation of oligodendrocytes. This finding is significant because it demonstrates that manipulating trained immunity in innate immune cells in vivo can reverse a detrimental process. Epigenetic reprogramming likely shifts microglia toward a pro-regenerative phenotype, improving their ability to clear debris and support remyelination. Reversing the presumed trained immunity process in monocytes/macrophages could attenuate the hyper-inflammatory state and promote a pro-regenerative phenotype, supporting effective remyelination within lesions.

Clinical trials have investigated BCG’s role in MS, given its role as an immunomodulator. A 1999 study reported reduced MRI activity in 14 RRMS patients, with no major adverse effects [59]. In a placebo-controlled study in patients with CIS patients, BCG reduced new T1-hypointense lesions in six months, decreased relapses after 18 months, and lowered progression to definite MS over five years [60]. Additionally, a strong tuberculin skin test post-vaccination was linked to reduced MS risk over 30 years [61]. However, a 2022 population-based study in Quebec found no significant association, indicating controversy and the need for more research [62].

Likewise, modulating macrophages’ metabolism may offer a viable strategy to mitigate the enhanced pro-inflammatory state associated with trained immunity. Wang et al. demonstrated that upregulating oxidative phosphorylation and decreasing glycolysis in mouse macrophages reduces the production of pro-inflammatory cytokines, thereby inhibiting disease progression in a murine model [63]. Metabolic rewiring through glucocorticoid (GC) treatment can promote anti-inflammatory effects in immune cells by redirecting their energy use. GCs, known to activate glucocorticoid receptors (GRs) that function as transcription factors, were found to induce the release of pyruvate dehydrogenase (PDH). This triggers mitochondrial pyruvate uptake, leading to the production of itaconate via the TCA cycle, which activates the anti-inflammatory transcription factor NRF2. This process reduces the expression of inflammatory genes such as *IL-1β*, *IL-6*, *TNF*, and *Nos2* in inflammatory macrophages. Furthermore, GCs inhibited severe LPS-induced inflammation in mice and showed beneficial effects in human and mouse models of rheumatoid arthritis—an autoimmune disease—and asthma [64]. Metabolism-associated modifications, such as lactylation have emerged as key regulators. Studies showed that lactylation of pyruvate kinase M2 (PKM2)—a glycolytic enzyme responsible for pyruvate formation—dampens the inflammatory metabolic adaptations in pro-inflammatory macrophages [65], while histone lactylation boosts reparative gene activation after myocardial infection [66]. Together, these studies underscore the idea that targeting metabolic pathways in macrophages can recalibrate their immune responses, potentially mitigating the chronic inflammation associated with trained immunity.

Another possibility for recalibrating trained immunity in MS monocytes/macrophages is to induce tolerance in these cells [67]. This is particularly relevant because autoimmunity is associated with a failure of self-tolerance, which is the immune system’s ability to recognize self-produced antigens as harmless, thereby preventing an autoimmune response [68].

Helminths were proposed as potential immunomodulators. For instance, Kooij et al. demonstrated that *Trichuris suis* shifts human monocytes toward a tolerance response, reducing their ability to migrate through the blood–brain barrier (BBB) in vitro [69]. Similarly, treatment with *Fasciola hepatica* total extract induces an anti-inflammatory form of trained immunity in mice—now recognized as tolerance [70]—dampening antigen-presenting cell function and T cell activity, which led to a milder course in EAE mice [71]. Additionally, helminth-derived extracellular vesicles were shown to reprogram HSCs and their progeny, delaying clinical progression and lessening EAE severity [72]. These findings highlight the potential of helminth-derived therapies as a novel strategy to modulate immune responses and combat chronic inflammation in MS.

Although these studies do not specifically address the reversal of trained immunity in pathological contexts, they do highlight potential research directions for this objective. For instance, altering cellular metabolism or promoting tolerance may reprogram innate immune responses, which are fundamental aspects of trained immunity.

## 6. Conclusions

In summary, manipulating trained immunity represents a novel strategy for developing MS treatments by modulating the innate immune response. These strategies not only reduce pathological inflammation but also promote tissue repair and remyelination, offering promising avenues to attenuate disease progression. Future therapies that target these mechanisms could ultimately recalibrate the dysregulated immune memory in MS, paving the way for more effective long-term treatment solutions.

## Figures and Tables

**Table 1:** Alterations in monocyte and macrophage phenotypes and functions in MS patients.

Aspect	Alteration in MS	Refs.
Monocyte subset frequencies	↑ Non-classical CD14^+^CD16^++^ monocytes in blood and CSF	[24,25,26]
Differentiation propensity	CD14^+^CD16^−^ monocytes from MS patients more readily differentiate into CD16^+^ macrophages	[30]
Activation marker expression	↑ CD40, CD86, HLA-DR, CD64, CCR2 (predominantly on CD16^+^ cells)	[28]
Pro-inflammatory cytokines	↑ IL-6, IL-12 in monocytes; ↑ IL-1β, TNF, NLRP3 in PBMCs; ↑ CCL4 in macrophages	[24,30,40,41]
Anti-inflammatory cytokines	↓ CCL17 in MS macrophages under homeostatic/pro-regenerative stimuli	[30]
Glycolytic activity	↑ Glycolysis in PBMCs and monocytes/macrophages (RRMS patients and EAE model)	[31]
Mitochondrial metabolism	↓ TCA cycle, ↓ fatty-acid oxidation, ↓ electron-transport chain activity, ↓ NAD^+^ levels in untreated MS macrophages	[30]
AAA (aromatic AA) pathways	↓ Reductive (e.g. indolelactate, phenyllactate), ↑ Oxidative metabolites (e.g. p-cresol sulfate, indoleacetate); overall lower AAA pathway activity correlating with disability scores	[32]
Genetic/epigenetic regulation	Enrichment of MS-risk variants in monocyte/microglia regulatory regions; altered chromatin accessibility affecting genes like NFKB1, STAT3, IRF8	[33,34]
lncRNA expression	↓ NF-κB–inhibiting lncRNAs: MKI67IP and HNF1A-AS1	[38]
miRNA expression	↑ miR-155, ↓ miR-223 (skewing toward pro-inflammatory polarization)	[39]

↑ = increased/upregulated and ↓ = decreased/downregulated in MS vs. HCs. The illustration uses yellow to depict the expression of cell surface markers or cytokine chemokines. Production metabolic alterations are shown in green, while genetic and epigenetic changes are underlined in blue.

## Data Availability

No new data were created.

## Abbreviations

AAA	aromatic amino acid
ARMSS	Age-Related Multiple Sclerosis Severity
BBB	blood–brain barrier
BCG	Bacille Calmette-Guérin
CIS	clinically isolated syndrome
CNS	central nervous system
CSF	cerebrospinal fluid
CMV	cytomegalovirus
EAE	experimental autoimmune encephalomyelitis
EBNA	EBV nuclear-antigen
EBV	Epstein-Barr virus
EDSS	Expanded Disability Status Scale
GC	glucocorticoid
GR	glucocorticoid receptor
HC	healthy control
HERV	human endogenous retrovirus
HK2	hexokinase 2
HSC	hematopoietic stem cell
LncRNA	long non-coding RNA
MiRNA	microRNA
MS	multiple sclerosis
OPC	oligodendrocyte precursor cell
OXPHOS	oxidative phosphorylation
PBMC	peripheral blood mononuclear cell
PDH	pyruvate dehydrogenase
PFKP	phosphofructokinase, platelet type
PKM2	pyruvate kinase M2
PPMS	primary progressive MS
RRMS	relapsing-remitting MS
SNP	single nucleotide polymorphism
TCA	tricarboxylic acid
UMLILO	upstream master lncRNA of the inflammatory chemokine locus
2DG	2-deoxy-D-glucose
